# Bad Breath

**Published:** 1859-12

**Authors:** 


### BAD BREATH.

If when the face is brought near anothers', the lips are kept firmly closed there is no bad breath, that which comes from the nose being not perceptibly disagreeable.

Much of the disagreeable odor of a late meal may be avoided if the teeth and mouth are well rinsed with warm water, and the tooth brush is passed across the back part of the tongue.

In some persons, a fœtor of breath and of the feet alternate. In others, both are present at the same time.

A fœtid effluvia arises usually, if not always from three causes; first it is hereditary, being connected with a scrofulous taint; second, it arises from a want of personal cleanliness; third, it attends a disordered stomach. The second and third suggest their own remedies. The first is a grievous and mortifying misfortune to all sensitive minds, but it may be remedied to a very considerable extent, by persistent habits of strict personal cleanliness, by large out door activities, personal regularities, and the temperate use of plain substantial food, carefully avoiding all gross and rancid articles of diet, suet, cheese, pies, puddings, smoked and fried meats, fish and the like, using often and efficiently the vapor or warm bath, with soap and plentiful friction.

